# Pathophysiological Role of Vitamin D Deficiency in Down Syndrome: Insights into Metabolic Dysfunction and Sarcopenia

**DOI:** 10.3390/ijms262110756

**Published:** 2025-11-05

**Authors:** Maria Stella Valle, Cristina Russo, Sofia Surdo, Maria Teresa Cambria, Mariachiara Campanella, Michele Tuttobene, Lucia Malaguarnera

**Affiliations:** 1 Laboratory of Neuro-Biomechanics, Section of Physiology, Department of Biomedical and Biotechnological Sciences, University of Catania, 95123 Catania, Italy; 2 Section of Pathology, Department of Biomedical and Biotechnological Sciences, School of Medicine, University of Catania, 95123 Catania, Italy; cristina.russo@unict.it (C.R.); lucmal@unict.it (L.M.); 3 Italian Center for the Study of Osteopathy (CSDOI), 95124 Catania, Italy; s.surdo98@yahoo.com; 4 Section of Biochemistry, Department of Biomedical and Biotechnological Sciences, School of Medicine, University of Catania, 95123 Catania, Italy; mariachiara.campanella97@gmail.com; 5 ARNAS Garibaldi Hospital, 95123 Catania, Italy; tuttobenemichele@tiscali.it

**Keywords:** Down syndrome, vitamin D, striate muscle, sarcopenia, diabetes mellitus, metabolic dysregulation, irisin, ghrelin

## Abstract

People with Down syndrome represent a highly vulnerable population, frequently showing vitamin D deficiency together with an elevated risk of metabolic and neuromuscular dysfunction. This susceptibility derives from several factors, including muscular hypotonia, excess body weight, thyroid abnormalities, and immune dysregulation. The coexistence of these conditions compromises bone and muscle health, increases cardiometabolic risk, and reduces motor abilities and coordination, thereby predisposing individuals to falls, sarcopenia, sarcopenic obesity, and long-term disability. Vitamin D, traditionally known for its essential role in bone health, is now recognized as a pleiotropic hormone regulating immune responses, metabolic balance, and muscle performance. Its deficiency is increasingly linked to obesity, insulin resistance, diabetes mellitus, dyslipidemia, and metabolic syndrome. These adverse outcomes are mediated through mechanisms involving chronic inflammation, oxidative stress, mitochondrial impairment, and disrupted adipokine signaling. This review integrates current molecular, cellular, and clinical evidence on the multifaceted actions of vitamin D in Down syndrome. Particular emphasis is placed on its effects on insulin signaling, adipose tissue metabolism, inflammatory regulation, and muscle strength. Finally, vitamin D is discussed as a biomarker and therapeutic target to guide personalized interventions aimed at improving metabolic health, maintaining muscle function, and promoting long-term independence in this high-risk population.

## 1. Introduction

Down syndrome (DS), also known as trisomy 21 for additional copying of chromosome 21—is one of the most common chromosomal abnormalities causing intellectual disability. DS occurs in approximately one in 700 live births [1]. Clinical features of DS include delays in physical and motor development and cognitive impairment. Individuals with DS throughout their lives may be affected by a wide range of comorbidities including congenital heart defects, gastroesophageal reflux disease, chronic constipation, muscular hypotonia, hearing and vision disorders, autoimmune diseases such as Hashimoto’s thyroiditis, celiac disease and type one diabetes [2]. Individuals with DS often have chewing and swallowing difficulties, food selectivity, and delayed acquisition of independent food capabilities. These difficulties cause a propensity for both nutritional deficiencies, but also overweight, obesity, insulin resistance and cardiometabolic risk. For example, among young people with DS, 64% have a Body Mass Index in the ≥85° percentile. In these subjects there is a significantly higher prevalence of prediabetes, dyslipidemia and systemic inflammation compared to peers with typical development [3,4]. Likewise, adults with DS often show abdominal obesity and increased insulin resistance [5]. In fact, Individuals with DS frequently present with muscular hypotonia, ligamentous laxity, and diminished muscle strength, features that compromise balance, coordination, and postural stability. These motor deficits have been documented in multiple observational and biomechanical studies: children and adolescents with DS display musculoskeletal anomalies, hypotonia and joint laxity [6], contributing to instability in posture and gait [7]. Articles report proprioceptive impairment, reduced strength, and delayed motor performance across developmental stages [8], while biomechanical analyses confirm excessive postural sway and coordination deficits during balance-challenging tasks [6,9].

In subjects with DS, musculoskeletal abnormalities may evolve into sarcopenia at an accelerated rate, and to a greater extent than in their peers without DS [10]. Sarcopenia is a condition characterized by the progressive reduction of muscle strength and muscle mass associated with functional limitations and physical disability in activities of daily living. Sarcopenia has been primarily associated with aging [11]. However, sarcopenia may also be due to other factors such as poor nutrition, physical inactivity and chronic diseases [12], all of which are problematic for special populations such as adults with DS. Additionally, young adults with DS have high adiposity associated with sarcopenia, a condition known as sarcopenic obesity (SO).

SO appears to be generated by higher levels of metabolic disorders and accompanied by an increased risk of mortality compared to obesity or sarcopenia alone [13,14].

The pathophysiological mechanisms of sarcopenia and SO are caused by inflammatory status, disruption of protein balance, mitochondrial dysfunction, genomic and epigenetic instability, abnormal intercellular signaling, dyslipidemia and dysregulation of nutritional signaling [15]. Although multiple factors can be attributed to sarcopenia and SO, such as lack of physical activity and poor nutrition, accelerated aging of individuals with DS could also play a role. Likewise, congenital conditions such as hypotonia could lead to the development of sarcopenia and sarcopenic obesity in this special population [16]. Musculoskeletal function is also strongly influenced by nutritional and genetic factors [17].

Some studies have suggested the importance of an optimal state of vitamin D levels in subjects with DS, pointing out that vitamin D deficiency (VDD) could represent a marker for several comorbidities typical of DS subjects such as obesity, diabetes, cardiovascular risk, hypertension, dyslipidemia, muscle weakness and sarcopenia [4,18,19].

Vitamin D, being a regulator of Ca^2+^, preserves the health of the skeleton. Its deficiency is linked to bone diseases such as rickets and osteoporosis. However, vitamin D can show important immunoregulatory, anti-inflammatory and antioxidant effects.

In recent decades, much experimental evidence shows a key molecular role for vitamin D in metabolic function and skeletal muscle function, and its deficiency is often associated with pain, muscle weakness and sarcopenia. VDD, commonly seen in DS, has been shown to exacerbate insulin resistance, metabolic and muscle dysfunction, while supplementation can improve these conditions in both human and experimental models [20]. Evaluation and optimization of vitamin D status in DS represents the dual opportunity to support neuromuscular function and metabolic health. In this review, we synthesize current knowledge on the multiple roles of vitamin D, emphasizing its impact on metabolic regulation and the musculoskeletal axis (muscle performance, functional mobility and sarcopenia), in individuals with DS. By integrating mechanistic insights and clinical evidence, we highlight the potential of vitamin D as both a biomarker and therapeutic target and discuss opportunities for personalized strategies to improve health outcomes in this high-risk population.

## 2. Sarcopenia and SO: Pathophysiological Similarities and Differences

Sarcopenia and SO share some commonalities but also exhibit significant differences in pathophysiological characteristics. The most relevant differences between sarcopenia and SO were found in lipid metabolism and fat–muscle metabolic interaction. Differences in inflammatory characteristics between sarcopenia and SO were also detected, which mainly consist of a greater increase in C-reactive protein (CRP), in SO. Conversely, in sarcopenia, the levels of pro-inflammatory cytokines such as TNF- α and IL-6 are primarily elevated [21]. In SO, chronic inflammation causes the increase of hepatic acute phase proteins such as CRP and inhibition of protein synthesis such as albumin, reflecting alterations in muscle–liver metabolic interactions. These alterations aggravate the degradation of muscle proteins and their functional loss. In addition, leukocytosis found in SO is strongly related to the interaction between adipose tissue and immune cells [22]. In fact, the increase in visceral fat induces inflammation and recruitment and infiltration of monocytes into adipose tissue. Monocytes, differentiating into dendritic cells, produce IL-23, favoring the extramedullary expansion of neutrophils [23]. Instead, specific increases in TNF- α and IL-6 in sarcopenia levels reflect a muscle-autonomous inflammatory pathway [24]. Muscle atrophy generates mitochondrial dysfunction with the release of mitochondrial DNA and the activation of the NLRP3 inflammasome, favoring a significant production of TNF-α. TNF-α, in turn, by activating the NF-κB and JNK signaling pathways, which promote the degradation of muscle proteins and the suppression of muscle synthesis, aggravates muscle atrophy [25,26]. Meanwhile, IL-6 limits the differentiation capacity of muscle stem cells through SOCS3 feedback and, in synergy with TNF-α, weakens muscle anabolism and blocks energy homeostasis, causing catabolism and muscle atrophy [27]. The interaction between IL-6 and TNF-α highlights the high sensitivity of muscle to inflammatory signals and reflects the intrinsic phenotype of inflammaging [28]. From the evidence stated so far, it can be deduced that sarcopenia and SO could be determined by specific pathways in chronic inflammation. In fact, at the molecular level sarcopenia is considered a syndrome of protein degradation and chronic inflammation, while SO is classified as a metabolic inflammatory entity mediated by the fat–liver axis. Specifically, visceral adipose tissue leads to CRP production through IL-6 independent pathways, directly activating CRP transcription in hepatocytes via the NF-κB pathway [29]. In addition, free fatty acids released from visceral fat improve hepatocyte sensitivity to low IL-6 levels through the TLR4-MyD88 pathway, causing a “synergistic amplification effect” on CRP production [30]. Adipose tissue is an active endocrine organ that, by secreting hormones and cytokines influences systemic inflammatory status. In particular, adipocytes or infiltrating macrophages in adipose tissue induce adipokines and proinflammatory cytokines, which, in turn, induce the production of CRP in the liver. Bioactive substances secreted by adipose tissue, such as leptin, participate in metabolic regulation through signaling pathways including JAK2/STAT3, while inflammatory factors such as TNF-α can suppress gene expression of various proteins [31]. Regarding tissue interactions, sarcopenia is increasingly recognized as a muscle-centered disorder that also affects and is affected by both the adipose and skeletal systems. Chronic inflammation and mitochondrial dysfunction in skeletal muscle alter myokine secretion, reducing factors such as irisin and myostatin that normally mediate communication between muscle and bone. This disrupted crosstalk contributes to reduced osteoblast activity, impaired bone remodeling, and the onset of osteosarcopenia [32]. Additionally, muscle-derived IL-6 and TNF-α act systemically to enhance osteoclast differentiation, further promoting bone resorption and skeletal fragility [33]. Thus, sarcopenia involves not only a local muscle-autonomous inflammatory phenotype but also a systemic endocrine imbalance that extends to adipose and bone tissues. This muscle–bone–fat axis represents a critical determinant of age-related musculoskeletal decline, where chronic low-grade inflammation (“inflammaging”) perpetuates a self-sustaining cycle of catabolism and tissue dysfunction [34]. Regarding mitochondrial involvement in sarcopenia, mitochondrial heterogeneity in adipose tissue and muscle fat infiltration may result from selective atrophy of beige fat [35]. While SO shows typical white fat dysfunction combined with increased resistance to FGF21, leading to a disturbance of adipose tissue’s ability to store and metabolize triglycerides [36] (Table 1).

**Figure 1:**
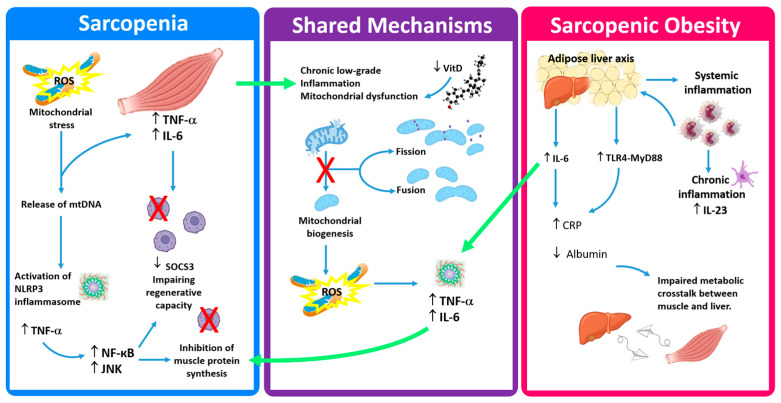
Distinct and shared inflammatory and metabolic pathways in sarcopenia and sarcopenic obesity (SO). In sarcopenia, mitochondrial stress and mtDNA release activate the NLRP3 inflammasome and the TNF-α/IL-6 axis, leading to NF-κB/JNK-mediated inhibition of muscle protein synthesis and reduced regenerative capacity. Shared mechanisms include chronic low-grade inflammation, mitochondrial dysfunction, and vitamin D deficiency, which impair mitochondrial biogenesis and exacerbate ROS production. In SO, inflammation originates from the adipose–liver axis, where IL-6 and TLR4–MyD88 signaling increases promote CRP synthesis and albumin reduction, while IL-23-driven systemic inflammation disrupts muscle–liver metabolic crosstalk. Arrows illustrate the main molecular mechanisms underlying sarcopenia and SO (blue arrows show interaction; green arrows show the common interaction; red crosses represent inhibition effect). **Abbreviations**: CRP = C-Reactive Protein; IL-6 = interleukin-6; IL-23 = interleukin-23; NLRP3 = NOD-, LRR- and pyrin domain-containing protein 3; ROS = radical oxygen species; SOCS3 = Suppressor Of Cytokine Signaling 3; TLR4-MyD88 = Toll-like receptor 4-MyD88; TNF-α = tumor necrosis factor-α; VitD = vitamin D.

At the endocrine level, VDD is a shared feature of sarcopenia and SO, suggesting that both conditions involve endocrine dysregulation and, albeit in different ways, impairment of skeletal muscle mitochondrial function (Figure 1).

## 3. Molecular Mechanisms Linking Vitamin D to Metabolic and Muscle Function

Vitamin D is a prohormone available in two active forms: vitamin D3 (cholecalciferol) and D2 (ergocalciferol). Both vitamins D3 and D2 follow the same metabolic pathway for the synthesis of their biologically active form. Vitamin D can be obtained either by epidermal synthesis of UVB rays or by dietary intake. UVB rays from sunlight activate the photo-isomerization of 7-dihahydrocholesterol into vitamin D [37]. The first metabolic phase that occurs in the liver leads to the transformation of vitamin D into 25-hydroxyvitamin D by the action of (CYP2R1). 25-Hydroxyvitamin D is the main circulating form of vitamin D and is used to clinically evaluate vitamin D status. 25-Hydroxyvitamin D binds to a protein carrier, the vitamin D binding protein. The resulting complex is transferred to the kidney, where it undergoes a final hydroxylation step catalyzed by CYP27B1 to form the biologically active metabolite, 1α,25-dihydroxyvitamin D or calcitriol [38]. Thus, vitamin D regulates insulin secretion and sensitivity by binding to vitamin D receptor (VDR) in pancreatic β cells, adipocytes, and skeletal muscle [39]. Deficiency since it can amplify insulin resistance and glucose intolerance is associated with the susceptibility of onset of diabetes mellitus, increasing systemic inflammation through elevated pro-inflammatory cytokines such as IL-6, TNF-α and TNF-β and increased CRP [40]. VDR is also expressed in skeletal muscle [41], where it regulates genes involved in contractility, mitochondrial biogenesis, protein synthesis, and energy metabolism [42]. Interestingly, 1 a-hydroxylase (CYP27B1), is also expressed in muscle, allowing local transformation from inactive to active vitamin D. Thus, the action exerted by vitamin D in muscle is due to the expression of vitamin D system-related proteins, including VDR, CYP27B1 and CYP24A1 in both skeletal muscle cells and tissues [43]. Furthermore, VDR, CYP27B1 and CYP24A1 are expressed, after injury, in the skeletal muscle stem cells (i.e., satellite cells) of the regenerating muscle, confirming the direct involvement of vitamin D in facilitating muscle regeneration and supporting muscle volume. Furthermore, vitamin D influences mitochondrial function and oxidative stress in muscle tissue, thus supporting energy metabolism and reducing the generation of reactive oxygen species. Recent evidence highlights its antioxidant benefits for improving muscle performance [44], as well as its synergistic role with bioactive molecules such as resveratrol in promoting mitochondrial health and counteracting sarcopenia [45].

## 4. Mechanisms Employed by Vitamin D in Slowing the Evolution of Sarcopenia

Vitamin D regulates skeletal muscle function through genomic and non-genomic effects. Genomic effects involve the interaction between vitamin D, VDR and specific nuclear receptors that control gene transcription. The non-genomic effects are due to the interaction between vitamin D and its non-nuclear receptors, activating intracellular signal transduction through different pathways [38,39,44]. Through genomic regulation and gene signaling pathway, the active form of vitamin D is able to influence the proliferation and differentiation of muscle cells [3,4]. In skeletal muscle derived stem cells vitamin D regulates cellular differentiation by inducing myogenic transcription factors including fetal myosin, neural cell adhesion molecule, insulin-like growth factor-I, fibroblast growth factor and myogenic differentiation protein [45]. Furthermore, in mouse C2C12, skeletal muscle cells vitamin D induces cell proliferation through up-regulation of follistatin and insulin-like growth factor [46]. Since vitamin D regulates muscle regeneration by promoting an increase in the cross-sectional area of skeletal muscle fibers by arresting the cell cycle, it could effectively influence muscle regeneration in subjects with DS [8,47,48]. Furthermore, it suppresses the expression of myostatin, a negative regulator of muscle, preventing muscle degeneration and improving contractile filaments and muscle strength [49]. Experimental tests in a mouse model have shown that VDR is important for the reduction of lean mass, sarcopenia, reduced grip strength and physical performance, confirming that the vitamin D-VDR interaction plays an important role in the physiology of skeletal muscle [50]. Skeletal muscles exert metabolic activity and secretory function. Irisin is a myokine released from skeletal muscles after proteolysis of the membrane protein fibronectin type III domain containing 5 (FNDC5), acting as a signaling protein [51]. In muscle, the expression of FNDC5 is induced by a PGC1 alpha dependent pathway. Additionally, irisin is regulated by the activation of p38MAPK [38].

**Figure 2:**
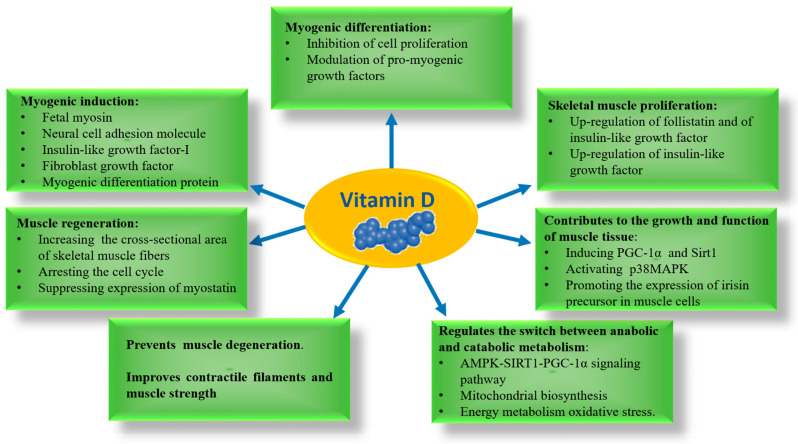
Schematic representation of the molecular mechanisms by which vitamin D modulates skeletal muscle physiology. Through these pathways, vitamin D contributes to muscle growth, regeneration, and metabolic homeostasis.

Irisin is related to the activity of subcutaneous white adipose tissue. It induces uncoupling protein 1 (UCP1) expression and stimulates brown adipocytes in white adipose tissue depots through white fat browning [51]. Irisin also promotes thermogenesis, which is related to insulin sensitivity, body weight, and glucose metabolism [52]. Several investigations suggested that decreased irisin levels are associated with the development of insulin resistance and its related diseases, such as T2DM and MetS. In fact, the association between irisin and glucose homeostasis is related to increased fatty acid oxidation and consumption of glucose via the adenosine monophosphate activated protein kinase (AMPK) signaling pathway in a diabetic murine model [53]. At high levels, irisin regulates the expression of inflammatory cytokines such as IL-1β and TNF-α [54]. Regarding the relationship between vitamin D and irisin, both molecules are essential regulators of the musculoskeletal apparatus and energetic homeostasis. Interestingly, vitamin D contributes to the growth and function of muscle tissue through interactions between VDR and PGC-1α and activation of p38MAPK in muscle with a mechanism similar to irisin [38]. In addition, vitamin D treatment promotes the expression of irisin precursor in muscle cells only if an integral expression of Sirt1 occurs, which in turn, is able to activate AMPK. AMPK is a core component of the AMPK-SIRT1-PGC-1α signaling pathway that regulates the switch between anabolic and catabolic metabolism. It is also well known that AMPK-SIRT1-PGC-1α signaling pathway acts as an energy sensing network that is crucial for mitochondrial biosynthesis, energy metabolism and oxidative stress. Being the main regulators of the oxidative capacity of muscle fiber and mitochondrial biogenesis, both AMPK and Sirt1 induce transcription of PGC-1α, which in turn helps various transcription factors to regulate the mitochondrial content of the tissue [55,56]. Notably, vitamin D supplementation induces expression of FNDC5 in the skeletal muscle of diabetic patients [57]. Many mechanistic linkages described herein are supported by data from general or diabetic populations that would deserve investigations in subjects with DS (Figure 2).

## 5. Impact of Vitamin D Deficiency on Muscle Performance of DS

In individuals with DS, who frequently present with hypotonia, ligamentous laxity, and reduced muscle mass, vitamin D deficiency amplifies existing neuromuscular deficits [58]. This molecular interplay manifests clinically as decreased gait stability, impaired balance, increased risk of falls, and limited functional mobility. Additionally, vitamin D modulates insulin signaling and glucose uptake in muscle and adipose tissue, linking muscular and metabolic pathways. By regulating inflammatory mediators and oxidative stress, vitamin D helps maintain cellular homeostasis, supporting both metabolic health and neuromuscular function.

In DS, pathways involved in oxidative stress and mitochondrial dysfunction are often already compromised due to trisomy 21 and related genetic effects. For example, overexpression of genes located on chromosome 21, such as SOD1 and APP, leads to oxidative stress and mitochondrial dysfunction, as described in systematic reviews [45]. Further mechanisms involve mitochondrial imbalance studies (RCAN1, DYRK1A, NRIP1, etc.) in DS [48]. Beyond oxidative and mitochondrial stress, individuals with DS exhibit early glial activation and chronic low-grade neuroinflammation, that closely resemble Alzheimer-like neuropathology. Among the most relevant neuroinflammatory markers, chitinases, particularly chitotriosidase (CHIT-1) and chitinase-3-like protein 1 (CHI3L1/YKL-40), are secreted by activated microglia and astrocytes and have been identified as cerebrospinal fluid biomarkers of neurodegeneration in adults with DS [59,60]. Furthermore, CBS hyperactivity in DS cells has been shown to suppress oxidative phosphorylation and ATP production, through excessive generation of H_2_S [49]. Similarly, fetal fibroblast studies indicate that mitochondria of individuals with DS show structural and functional abnormalities including complex I inhibition and elevated ROS levels [50]. In individuals with DS, who frequently present with hypotonia, ligament laxity and reduced muscle mass, vitamin D deficiency amplifies existing neuromuscular deficits [58], manifesting as impaired gait, balance and functional mobility. Additionally, vitamin D modulates insulin signaling and glucose uptake in muscle and adipose tissue, linking muscular and metabolic pathways. By regulating inflammatory mediators and oxidative stress, vitamin D helps maintain cellular homeostasis, supporting both metabolic health and neuromuscular function [61].

Maintaining adequate vitamin D levels can therefore support both metabolic homeostasis and neuromuscular function, mitigating functional decline, improving muscle strength and coordination, and reducing the risk of falls and slowing sarcopenic and SO evolution [62].

## 6. Vitamin D, Ghrelin and Metabolic Regulation in Down Syndrome

The concentration of vitamin D depends on several factors, including sunlight exposure, skin pigmentation, adiposity and dietary intake [18]; unfortunately, DS patients often develop nutritional disorders [18].

It has been reported that lower concentration of vitamin D is associated with lower ghrelin levels in older women and indirectly with nutritional status expressed by low albumin levels [63].

Vitamin D is able in stimulating the secretion of adipokines such as adiponectin, leptin and resistin and therefore affects the energy homeostasis of the body [64,65].

Ghrelin, a peptide hormone primarily secreted by the stomach, is known for its role in stimulating appetite, regulating energy homeostasis, and modulating glucose metabolism. Beyond its peripheral metabolic functions, ghrelin also acts at the central level, particularly within the hypothalamus and other brain regions, modulating synaptic plasticity, and neurogenesis. These central actions are essential for the regulation of appetite, reward pathways, and cognitive processes [66]. Additionally, ghrelin exerts potent anti-inflammatory effects beyond these functions, and influences lipid metabolism, making it a critical mediator of overall metabolic health [67]. In individuals with DS, who exhibit a higher prevalence of obesity, insulin resistance, and dyslipidemia compared to the general population, alterations in ghrelin signaling may contribute to their unique metabolic phenotype [68]. Several studies suggest that circulating ghrelin levels in DS may be dysregulated, although data remain limited and sometimes inconsistent, reflecting the complex hormonal and metabolic interactions in this population [68,69].

In the Ts65Dn mouse model of DS, reduced plasma ghrelin levels were observed alongside increased energy intake and a positive energy balance, leading to increased adiposity [70]. These changes were associated with fasting hyperglycemia and hypoinsulinemia, without significant alterations in leptin levels. Additionally, exaggerated leptin and resistin responses were observed after glucose loading, maintaining the hyperglycemic–hypoinsulinemic phenotype. Dysregulation of the adipose–insulin axis was accompanied by elevated circulating inflammatory markers, such as galectin-3 and HSP72, and reduced IL-6 levels [70]. These findings suggest that ghrelin dysfunction may contribute not only to hyperphagia and increased adiposity but also to impaired glucose homeostasis and a pro-inflammatory state, further increasing cardiometabolic risk [70].

The interaction between ghrelin and other metabolic pathways in DS is particularly relevant given the common endocrine and adipokine dysfunctions observed in this syndrome. Individuals with DS frequently exhibit altered leptin and adiponectin profiles, thyroid dysfunction, and chronic low-grade inflammation, all of which may interact with ghrelin signaling to exacerbate energy imbalance, insulin resistance, and weight gain [68]. Ghrelin dysregulation may therefore contribute simultaneously to hyperphagia, adiposity, and compromised glucose homeostasis, heightening overall cardiometabolic risk.

Importantly, ghrelin also affects muscle and neuromuscular function, which is already impaired in DS due to hypotonia and reduced muscle mass. By modulating energy availability and metabolic signaling in skeletal muscle, ghrelin can influence muscle strength, endurance, and functional mobility, potentially impacting physical performance and daily activity levels [71]. Understanding these mechanisms highlights the dual role of ghrelin in both metabolic and musculoskeletal regulation.

Elucidating the precise role of ghrelin in DS could inform targeted interventions, including pharmacological approaches to modulate ghrelin activity, dietary strategies to optimize satiety and nutrient intake, and exercise programs designed to improve energy balance and metabolic health. Integrating ghrelin assessments into broader biomarker panels, including 25-hydroxyvitamin D, adipokines, and inflammatory cytokines, could further support precision medicine strategies tailored to the metabolic and functional needs of individuals with DS.

## 7. Biomarkers and Personalized Vitamin D Therapy

The integration of biomarkers into clinical practice offers a promising avenue for personalizing vitamin D supplementation in individuals with DS. Although serum 25-hydroxyvitamin D [25(OH)D] remains the primary marker for assessing vitamin D status, evidence suggests that relying solely on this measurement may not fully capture the metabolic and neuromuscular responses in individuals with DS. For example, Stagi et al. [18] reported that children with DS exhibited insufficient vitamin D levels even after supplementation, indicating that 25(OH)D alone may not reflect true vitamin D status. However, the study does not provide standardized dosage recommendations but rather describes the supplementation protocols used for subjects who were receiving treatment. Specifically, for subjects with a serum 25(OH)D level below 10 ng/mL, an initial loading dose of 300,000 IU of oral vitamin D_3_ was prescribed as a single administration, followed by a maintenance dose of 800 IU/day or 25,000 IU every three weeks for one year [18]. Similarly, Boyd et al. [19] highlighted that low 25(OH)D, whose average level for individuals with DS was significantly lower than that of the controls, can impair neural proliferation and long-term neurodevelopment, emphasizing the need for more comprehensive biomarker monitoring in this population. Expanding the panel of biomarkers to include inflammatory cytokines (e.g., IL-6, TNF-α), leptin, adiponectin, myokines such as irisin, and functional assessments of muscle strength, tone, and coordination, can provide a more comprehensive understanding of an individual’s baseline status and potential response to therapy [72]. Adipokines are signaling proteins produced by adipose tissue that play significant roles in regulating inflammation and metabolism. Elevated levels of pro-inflammatory cytokines like IL-6 and TNF-α have been associated with obesity and related metabolic disorders, offering insights into the inflammatory status and metabolic health of individuals. Additionally, inflammatory cytokines produced by adipose tissue, especially visceral fat, can accelerate muscle catabolism and contribute to sarcopenic obesity, highlighting the importance of monitoring these markers in conjunction with neuromuscular assessments. Furthermore, leptin and adiponectin are involved in various processes including inflammation, energy metabolism, and insulin sensitivity, and their levels can reflect the functional status of adipose tissue, providing valuable information for personalized therapeutic strategies [72].

By combining biochemical, functional, and genetic data, clinicians can move toward a precision medicine approach in which supplementation is tailored to each patient’s unique characteristics. Continuous monitoring of these biomarkers enables dynamic dose adjustments, tracks improvements in insulin sensitivity, reduces systemic inflammation, and optimizes neuromuscular performance [73].

Furthermore, integrating biomarker-guided strategies with lifestyle interventions, such as structured physical activity programs and targeted nutritional optimization, can amplify the benefits of vitamin D therapy. This approach allows clinicians to not only enhance the effectiveness of supplementation but also anticipate and prevent metabolic and musculoskeletal complications. Ultimately, a biomarker-informed, precision medicine framework provides a powerful tool to improve functional outcomes, mobility, and quality of life in individuals with DS. By aligning therapeutic decisions with each patient’s specific physiological profile, this approach facilitates personalized interventions. Studies have demonstrated that monitoring plasma biomarkers can inform therapeutic strategies [74,75], leading to improved outcomes in DS. Additionally, the application of personalized medicine in DS underscores the significance of developing biomarkers to understand and manage co-morbidities, thereby optimizing care [68].

## 8. Knowledge Gaps

Despite growing recognition of vitamin D’s importance in DS, several critical gaps remain regarding optimal dosing, frequency, and duration of supplementation. Studies have shown that vitamin D deficiency is highly prevalent in this population, particularly in the presence of obesity and autoimmune comorbidities, yet standardized supplementation protocols are lacking [18]. Moreover, vitamin D deficiency in obese children has been associated with elevated circulating inflammatory mediators and reduced insulin sensitivity, but the long-term effects of supplementation on these parameters remain poorly characterized [76]. Vitamin D also plays a regulatory role in insulin sensitivity and lipid metabolism, suggesting that supplementation could influence the balance between pro- and anti-inflammatory cytokines. Nevertheless, evidence on the long-term impact of vitamin D supplementation on insulin sensitivity, lipid metabolism, inflammation, and muscle function in DS is still limited [77]. Genetic and endocrine factors unique to DS, including trisomy 21, associated alterations in VDR signaling, mitochondrial function, and oxidative stress, may influence responsiveness to vitamin D, yet their clinical significance is not fully understood. Furthermore, interactions with lifestyle factors, comorbidities, and coexisting endocrine disorders remain underexplored. Addressing these gaps is essential for developing evidence-based, personalized supplementation protocols.

## 9. Conclusions

Vitamin D plays a critical and multifaceted role in human physiology, influencing skeletal integrity, immune function, muscle performance, and metabolic homeostasis. Individuals with DS are particularly vulnerable to vitamin D deficiency due to factors such as reduced sun exposure, hypotonia, obesity, and altered metabolism, which in turn exacerbate their predisposition to metabolic disorders, including insulin resistance, dyslipidemia, and diabetes mellitus.

Adequate vitamin D status may mitigate these risks by improving insulin secretion and sensitivity, modulating chronic low-grade inflammation, and supporting healthy adipose tissue function. Moreover, by enhancing muscle strength and neuromuscular coordination, vitamin D can indirectly contribute to improved functional mobility and reduced risk of falls, addressing both metabolic and musculoskeletal challenges in DS.

While supplementation shows considerable promise, further research is needed to establish optimal dosing, assess long-term metabolic and musculoskeletal benefits, and develop individualized therapeutic strategies. Integrating vitamin D optimization with lifestyle interventions, such as tailored nutrition and physical activity programs, offers a comprehensive approach to improve health outcomes.

Overall, targeting vitamin D deficiency represents a promising avenue to enhance metabolic, skeletal, and neuromuscular health in individuals with DS, highlighting its potential as both a biomarker and a therapeutic tool in this high-risk population.

## 10. Future Directions

Future research should aim to define the optimal strategies for vitamin D supplementation in individuals with DS, integrating both metabolic and neuromuscular outcomes. Longitudinal studies and well-powered randomized controlled trials are needed to clarify the effects of supplementation on insulin sensitivity, lipid metabolism, systemic inflammation, muscle strength, coordination, and functional mobility. These studies should consider individual variability, including baseline vitamin D status, body composition, genetic differences affecting vitamin D receptor signaling, and coexisting medical conditions, supporting a precision medicine approach.

A critical avenue for investigation involves the development and validation of composite biomarker panels. Combining 25(OH)D measurements with inflammatory cytokines, adipokines, and functional muscle parameters can enable more accurate monitoring of therapeutic response and guide individualized dosing regimens. Research should also explore how vitamin D supplementation interacts with lifestyle interventions, such as structured exercise programs and dietary optimization, to maximize both metabolic and neuromuscular benefits.

Finally, identifying predictive biomarkers and early indicators of clinical improvement can facilitate proactive, preventive care. By focusing on these pathways, future studies can translate the molecular and physiological potential of vitamin D into tangible clinical benefits, enhancing functional independence, reducing cardiometabolic risk, and improving overall quality of life in this vulnerable population.

## Figures and Tables

**Table 1:** Sarcopenia vs. Sarcopenic Obesity: pathophysiological characteristics.

Aspect	Sarcopenia	Sarcopenic Obesity	References
Inflammatory profile	↑ TNF-α, ↑ IL-6 → muscle-autonomous inflammation	↑ CRP, ↓ albumin → liver-mediated inflammation	[21]
Key inflammatory pathways	Muscle atrophy → mitochondrial dysfunction → release of mtDNA → activation of NLRP3 inflammasome → ↑ TNF-α (NF-κB, JNK activation) → protein degradation and reduced synthesis	Visceral fat → recruitment of monocytes → differentiation into dendritic cells → IL-23 production → neutrophil expansion; adipose tissue-immune cell interaction central	[23,25]
Cytokine effects	IL-6 + TNF-α synergy → impaired stem cell differentiation (SOCS3), reduced anabolism, blocked energy homeostasis → muscle catabolism and atrophy	IL-6-independent CRP induction in hepatocytes via NF-κB; free fatty acids (visceral fat) → ↑ IL-6 sensitivity (TLR4-MyD88) → amplification of CRP production	[24,28,29]
Tissue interactions	Muscle–bone–fat crosstalk impaired; reduced myokine (irisin, myostatin) signaling alters osteoblast activity and bone remodeling, promoting osteosarcopenia. Chronic inflammation and mitochondrial dysfunction drive intrinsic “inflammaging” phenotype.	Fat–liver axis dominant; adipose tissue acts as an endocrine organ secreting adipokines (e.g., leptin, TNF-α) that alter systemic inflammation and hepatic protein synthesis	[31,32,33]
Mitochondrial dysfunction	Muscle mitochondrial dysfunction; infiltration of fat into muscle; selective atrophy of beige fat contributes to heterogeneity	White fat dysfunction, ↑ FGF21 resistance → impaired triglyceride storage and metabolism	[25,35,36]

## Data Availability

No new data were created or analyzed in this study. Data sharing is not applicable to this article.
